# Necrotic erythema nodosum reaction associated with histological alterations of Lucio’s phenomenon^☆^

**DOI:** 10.1016/j.abd.2020.09.016

**Published:** 2022-01-04

**Authors:** Larissa Daniele Machado Góes, Patrícia Motta de Morais, Paula Frassinetti Bessa Rebello, Antônio Pedro Mendes Schettini

**Affiliations:** a Fundação de Dermatologia Tropical e Venereologia Alfredo da Matta, Manaus, AM, Brazil; b Department of Dermatopathology, Fundação de Dermatologia Tropical e Venereologia Alfredo da Matta, Manaus, AM, Brazil; c Department of Tropical Diseases, Fundação de Dermatologia Tropical e Venereologia Alfredo da Matta, Manaus, AM, Brazil

**Keywords:** Leprosy, Multibacillary leprosy, *Mycobacterium* infections, *Mycobacterium leprae*

## Abstract

Patients with lepromatous or borderline leprosy may present two types of vasculonecrotic reactions: Lucio’s phenomenon (LP) and necrotic erythema nodosum leprosum (nENL). These are serious conditions, which mostly lead to life-threatening infectious and thrombotic complications. The authors report the case of a patient with leprosy recurrence associated with an atypical type II reaction with LP characteristics on histopathology.

A 32-year-old man with a history of multibacillary leprosy under treatment for ten years with negative bacilloscopy at discharge reported the appearance of erythematous papules on the upper limbs. He used prednisone 40 mg/day on his own for five months and, when it was discontinued, pustules, vesicles, painful crusts and bullae with purulent content appeared on the plantar region (Figure 1, Figure 2), together with fever and general malaise. Rapid tests for HIV, syphilis and hepatitis B and C were negative. The patient showed infiltration of the ear pinna and edema of the extremities. The bacilloscopy of the ear pinna revealed a 3.0 index (fragmented and granular bacilli). Histopathological analysis showed epidermal ulceration and a dense inflammatory infiltrate in the dermis, consisting of foamy macrophages, lymphocytes and a high number of neutrophils affecting superficial and deep vessels, adnexa and small nerves (Figs. 3A and 3B). The deep dermis vessels walls were permeated by neutrophils and there was leukocytoclasia. Wade staining showed intact and fragmented bacilli forming globi. Some bacilli were present in endothelial cells (Fig. 3C). Molecular analysis using the PCR technique did not show any drug resistance of *M. leprae* to dapsone, rifampicin and ofloxacin. The patient was submitted to a new regimen of multibacillary multidrug treatment (MB-MDT), prednisone 80 mg/day and systemic antibiotic therapy, with significant clinical improvement after three weeks.

**Figure 1:**
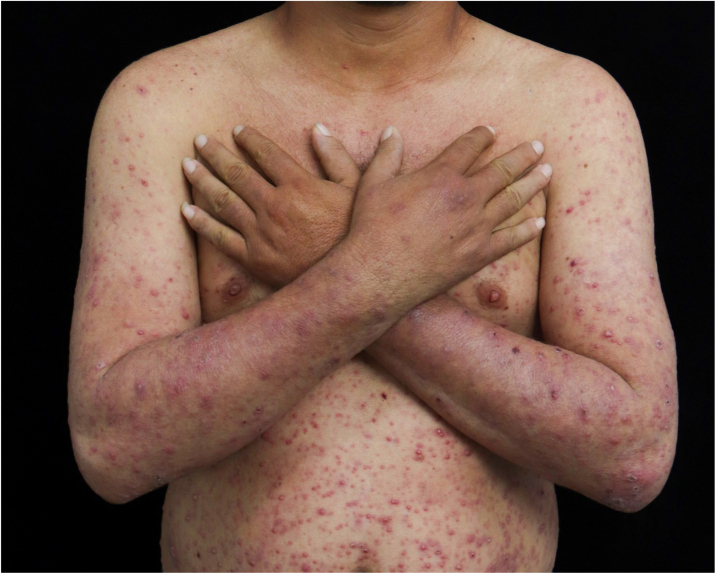
Presence of papules, pustules and crusts on the upper limbs and trunk.

**Figure 2:**
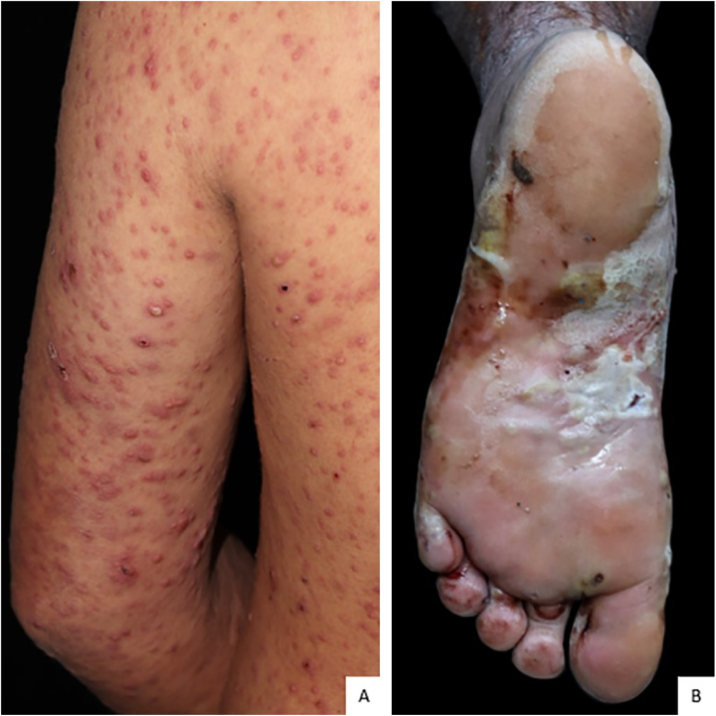
(A), Erythematous papules, pustules and crusts affecting the left upper limb. (B), Bullae with purulent content on the left plantar region.

**Figure 3:**
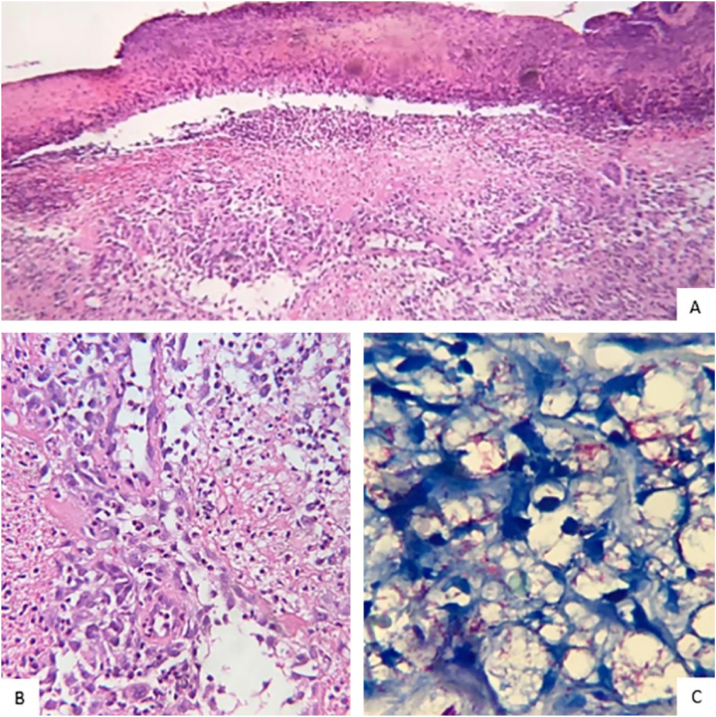
(A), Necrosis of the epidermis and dense inflammatory infiltrate in the dermis are observed (Hematoxylin & eosin, ×10). (B), Neutrophilic infiltrate on the wall and around the vessels (Hematoxylin & eosin, ×40). (C), Vacuolated macrophages containing large amounts of intact and fragmented bacilli (Wade, ×40).

Leprosy is a chronic granulomatous infectious disease with polymorphic manifestations caused by *Mycobacterium leprae*.^1^ Vasculonecrotic lesions in leprosy include Lucio’s phenomenon (LP) and necrotic erythema nodosum leprosum (nENL).^1^ These two reactive conditions can occur in patients with multibacillary or borderline multibacillary leprosy,^2^ although LP was originally described in patients with pure primitive multibacillary leprosy.^3^ In nENL, necrotic lesions appear over the nodules, usually after the beginning of the treatment,^4^ although they may occur before and after discharge. In LP, lesions develop over areas of infiltrated skin and progress to stellar scars.^4^ There is a risk of secondary bacterial infection and sepsis in both conditions, with a fatal outcome.^4^ Although nENL and LP are the results of immune-mediated phenomena,^4^ in LP, the action of the bacilli directly on the endothelium seems to be very important in the physiopathogeny.^5^ Both conditions respond favorably to corticosteroids and MB-MDT. Thalidomide is the treatment of choice for ENL^4^ and can be associated with corticosteroids. In the case reported here, the clinical changes favored nENL, but the histopathological finding of bacilli in the endothelial cells of the vessel wall is consistent with LP. As the diagnosis of leprosy in the public health system, is carried out essentially based on clinical findings, it is necessary that complementary tests, including histopathology, be available in the hierarchical health network so that conditions similar to the one described this report can be recognized early and adequately treated, preventing serious complications for the patient.

## Financial support

None declared.

## Authors' contributions

Larissa Daniele Machado Góes: Approval of the final version of the manuscript; design and planning of the study; drafting and editing of the manuscript; collection, analysis, and interpretation of data; effective participation in research orientation; intellectual participation in the propaedeutic and/or therapeutic conduct of the studied cases; critical review of the literature; critical review of the manuscript.

Patrícia Motta de Morais: Approval of the final version of the manuscript; design and planning of the study; drafting and editing of the manuscript; collection, analysis, and interpretation of data; effective participation in research orientation; intellectual participation in the propaedeutic and/or therapeutic conduct of the studied cases; critical review of the literature; critical review of the manuscript.

Paula Frassinetti Bessa Rebello: Approval of the final version of the manuscript; design and planning of the study; drafting and editing of the manuscript; collection, analysis, and interpretation of data; effective participation in research orientation; intellectual participation in the propaedeutic and/or therapeutic conduct of the studied cases; critical review of the literature; critical review of the manuscript.

Antônio Pedro Mendes Schettini: Approval of the final version of the manuscript; design and planning of the study; drafting and editing of the manuscript; collection, analysis, and interpretation of data; effective participation in research orientation; intellectual participation in the propaedeutic and/or therapeutic conduct of the studied cases; critical review of the literature; critical review of the manuscript.

## Conflicts of interest

None declared.
